# Helium-electrospray improves sample delivery in X-ray single-particle imaging experiments

**DOI:** 10.1038/s41598-024-54605-9

**Published:** 2024-02-22

**Authors:** Tej Varma Yenupuri, Safi Rafie-Zinedine, Lena Worbs, Michael Heymann, Joachim Schulz, Johan Bielecki, Filipe R. N. C. Maia

**Affiliations:** 1 Laboratory of Molecular Biophysics, Department of Cell and Molecular Biology, Uppsala University, Husargatan 3, Box 596, 75124 Uppsala, Sweden; 2 European XFEL, Holzkoppel 4, 22869 Schenefeld, Germany; 3 Institute of Biomaterials and Biomolecular Systems, University of Stuttgart, Pfaffenwaldring 57, Stuttgart, 70569 Germany; 4 Lawrence Berkeley National Laboratory, Berkeley, CA 94720 USA

**Keywords:** Imaging techniques, Biological physics, Single-molecule biophysics, Free-electron lasers, Imaging techniques, Biological physics, Single-molecule biophysics, Free-electron lasers

## Abstract

Imaging the structure and observing the dynamics of isolated proteins using single-particle X-ray diffractive imaging (SPI) is one of the potential applications of X-ray free-electron lasers (XFELs). Currently, SPI experiments on isolated proteins are limited by three factors: low signal strength, limited data and high background from gas scattering. The last two factors are largely due to the shortcomings of the aerosol sample delivery methods in use. Here we present our modified electrospray ionization (ESI) source, which we dubbed helium-ESI (He-ESI). With it, we increased particle delivery into the interaction region by a factor of 10, for 26 nm-sized biological particles, and decreased the gas load in the interaction chamber corresponding to an 80% reduction in gas scattering when compared to the original ESI. These improvements have the potential to significantly increase the quality and quantity of SPI diffraction patterns in future experiments using He-ESI, resulting in higher-resolution structures.

## Introduction

Current generation X-ray free electron lasers (XFELs) with their ability to produce highly intense X-ray pulses with durations of only a few tens of femtoseconds offer a powerful tool to image a wide variety of aerosolized particles at room temperature. Such high intensities on femtosecond time scales suggested that useful data could be collected from weakly scattering single proteins or viruses by outrunning radiation damage using the idea of “diffraction before destruction”^1^. Taking full advantage of this new capability of coherent diffractive X-ray imaging using single particles in the gas phase promises to not only deliver high-resolution structures but to extend the study towards ultrafast dynamics^2,3^, opening the door for pump-probe experiments on femto-and picosecond time scales. So far, single-particle imaging (SPI) experiments have been successfully performed by injecting the aerosolized sample into the X-ray interaction region using the “Uppsala”-injector^4^ on large biological samples (70-2000 nm) using gas dynamic virtual nozzles (GDVN’s) on viruses^5–10^, cell organelles^11^, whole cells^12^ and most recently on gold nanoparticles^13^ using electrospray ionization (ESI).

**Figure 1:**
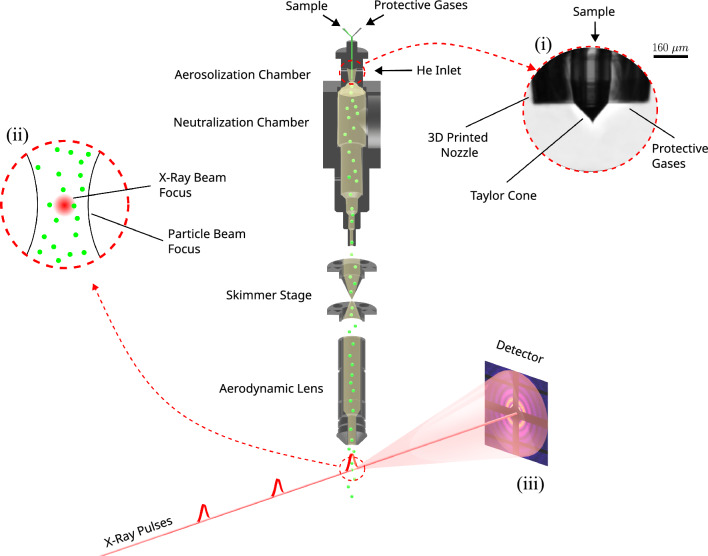
The schematic diagram details an experimental setup of a He-ESI-based aerosol injector designed for single particle imaging experiments at XFELs. This setup includes the He-ESI process illustrated at the top, which aerosolizes the sample. Subsequently, the aerosol beam is transported through the skimmer stages and the aerodynamic lens and eventually reaches the interaction chamber. Here, it intersects with the XFEL beam. The XFEL pulses scatter off the particles within the aerosol beam, generating diffraction patterns captured on the detector. (**i**) The Taylor cone, during standard operation of He-ESI. (**ii**) The interaction between a particle beam and an X-ray beam. (**iii**) Scattering pattern produced by a particle.

Gas phase injection^14,15^ via an aerodynamic lens stack (ALS) has gained substantial attention for its high scattering contrast, low background scattering compared to liquid sample delivery, capacity for high-rate data collection and wide sample compatibility. A typical experimental SPI layout can be found in^16^, and a modified experimental setup for He-ESI-based experiments is shown in Fig. 1. Particularly, ESI as a sample aerosolization method has proven effective due to its ability to produce small droplets, resulting in virtually contaminant-free sample delivery^17^. But even with the large pulse energies available at modern XFEL facilities, the diffraction patterns from small particles, such as single proteins or virus particles with sizes smaller than 50 nm have a very low signal-to-noise ratio preventing structure determination, despite computational efforts to reduce the noise^18^. A recent experiment on the GroEL complex from *E. coli* delivered using ESI highlights the challenge for small bioparticles: a high amount of background scattering from the N_2_ and CO_2_ in the interaction region^19^. The large gas background hampers the identification of signal from the sample of interest.

To obtain higher-resolution structures, a reduction of N_2_ and CO_2_ gas density in the interaction region to enhance the signal-to-noise ratio is necessary. In addition, improved particle throughput is needed to collect the several hundred thousand scattering patterns from identical particles needed to fill the 3D reciprocal space^20–22^. Therefore, improving sample delivery is one of the crucial factors in enabling high-resolution single-particle imaging at XFELs.

In this paper, we address these sample delivery challenges and present a modified ESI source, which we refer to as the Helium-electrospray (He-ESI). The main change is the addition of a 3D-printed nozzle, designed to reduce the N_2_ and CO_2_ consumption compared to the earlier setup (original ESI)^17^ while still maintaining stable sample delivery conditions. Helium (He) is introduced around the 3D-printed nozzle and serves as the main gas for particle transport. Our modifications lead to a lower N_2_ and CO_2_ use and a decrease of heavy gasses in the interaction region by  83%. We also demonstrate the successful use of the He-ESI with the “Uppsala”-injector and compare the performance with the original ESI in the injector setup. We observe an increase in injection yield which can be as high as a factor of  10 for the small biological particles.

Our He-ESI system shows great potential for SPI of small particles. The reduction in heavy-gas background effectively increases the signal-to-noise ratio. Furthermore, the use of He as the transport gas improves particle focusing in the “Uppsala”-injector, and enhances the throughput of particles into the interaction region. The ESI-setup developed here makes it possible to acquire millions of diffraction patterns with sufficiently low background, an important milestone on the way to high-resolution time-resolved 3D structures of isolated proteins and viruses using SPI.

## Methods and results

The experimental setup in this study consists of a modified version of the ESI introduced in^23^, the “Uppsala”-injector^4^ with a two-skimmer box setup, an optical scattering setup to detect the nanoparticles in the main chamber^24^ and a residual gas analyzer (RGA) (Extorr Inc., XT100M) to analyze the gas composition inside the chamber.

### Modified ESI source: He-ESI design

**Figure 2:**
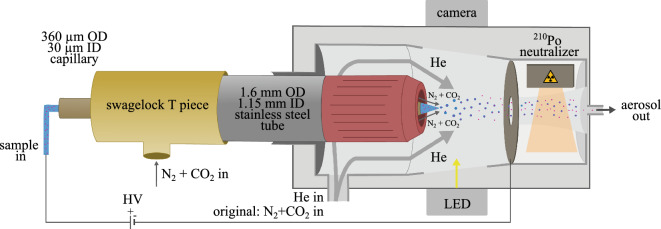
Schematic of the He-ESI. The modification of the ESI to operate with He includes a Swagelok T-piece, a stainless steel tube and the 3D-printed Uppsala nozzle. Liquid sample flows through the capillary and a stable Taylor cone is formed by applying a high voltage. In between the capillary and the inside of the nozzle a N_2_ and CO_2_ environment is formed with a combined flow rate of around 50 mL/min. Helium is introduced through the original gas inlet, surrounding the nozzle within the electrospray head and facilitating the flow of particles. The highly charged droplets pass through a Po-210 neutralizer. Then, the neutralized aerosol is exiting the electrospray head.

The modified ESI setup is shown in Fig. 2. It includes a 3D-printed nozzle (Uppsala nozzle) measuring 4.45 $\times$ 1.56 $\times$ 1.56 mm^3^ printed via two-photon polymerization in a liquid resin (UpPhoto) within 35 min using the NanoOne 3D printing system (UpNano). After printing, the nozzle was glued to a stainless-steel tube with an inner diameter (ID) of 1.15 mm using a standard two-component epoxy glue (Loctite power epoxy) and connected to the N_2_ and CO_2_ gas mixture line. To reduce the background scattering in SPI experiments, we replaced most of the N_2_ and CO_2_ used for particle transport with He. The gas inlet previously used for the N_2_ and CO_2_ gas mixture was used as the He inlet, as shown in Fig. 2. The Uppsala nozzle is designed to hold the silica fused capillary of 360 $\upmu$m outer diameter (OD) in the center of the nozzle as shown in Fig. 2. We reduced the consumption of N_2_ and CO_2_ by placing a 3D-printed structure around the capillary generating an N_2_ and CO_2_ atmosphere between the capillary and the nozzle and filling the rest of the ESI head with He.

An alternative 3D-printed nozzle design, referred to as the EuXFEL nozzle, follows the same principle of gas replacement but does not require the use of a fused silica capillary inside. Instead, it is entirely printed using the Nanoscribe Photonic Professional GT with IP-S photoresist. This design incorporated two capillary inlets: one with an ID of 40 $\mu$m for the sample and another with an ID of 180 $\upmu$m for protective gases (N_2_ and CO_2_). The dimensions of the EuXFEL nozzle are $1.4 \times 0.5 \times 1.2$ mm. Details on the EuXFEL nozzle can be found in the Supplementary Materials. Furthermore, the CAD models for both the Uppsala and EuXFEL nozzles are freely accessible and can be downloaded from our GitHub repository at (https://github.com/ytejvarma/Helium-nozzle) and (https://github.com/safirafie/ESDesign) respectively.

### Simulations of gas flow around the Taylor cone

In the electrospray process, a high voltage applied between the nozzle and a grounded orifice plate creates a cone-jet meniscus, known as a Taylor cone, which is formed when electric forces balance liquid surface tension^25,26^. The non-uniform electric field at the tip of the Taylor cone can induce a corona discharge at a high enough voltage. The electrons released from the ionized gas molecules neutralize the positive charges on the liquid surface which causes a collapse of the Taylor cone. Therefore, preventing corona discharge, while also minimizing the presence of heavier gases that contribute to higher background X-ray scattering, is essential for stable sample delivery in SPI experiments.

Considering the required voltage for Taylor cone formation, and the geometry around the nozzle, using only light gases like helium or hydrogen to support the electrospray is not feasible due to their low electric field threshold for corona discharge. The lightest gas mixture where we achieved stable operating conditions with the nozzle design presented here was: He at 1.2 L/min, N_2_ at 20 mL/min, and CO_2_ at 15 mL/min. Helium was the preferred light gas, considering hydrogen’s high flammability and explosive nature. N_2_ alone proved insufficient to prevent corona discharge while adding minimal amounts of CO_2_ insulates against the discharge.

Understanding the distribution of gas species around the Taylor cone in the He-ESI system is important for achieving stable sample delivery. Therefore, we performed finite element simulations using COMSOL Multiphysics^27^. We used the laminar flow interface and the transport of concentrated species interface and coupled these interfaces together through a multi-physics interface. The laminar flow interface allowed us to model the gas flow dynamics by computing the velocity and pressure fields of the gases. Concurrently, the transport of concentrated species interface was used to study gaseous mixtures by solving for the mass fractions of all participating species.

To monitor the risk of corona discharge around the Taylor cone, we calculated the fractional concentration of each gas, denoted as $x_{i}$ and defined as:

$$x_{i} = \frac{c_{i}}{c_{i} + c_{j} + c_{k} }$$

where $c_{i}$ symbolizes the molar concentration of the gas for which we are determining its fractional concentration, $x_{i}$. The $c_{j}$ and $c_{k}$ denote the molar concentrations of the remaining two gases in the mixture.

**Figure 3:**
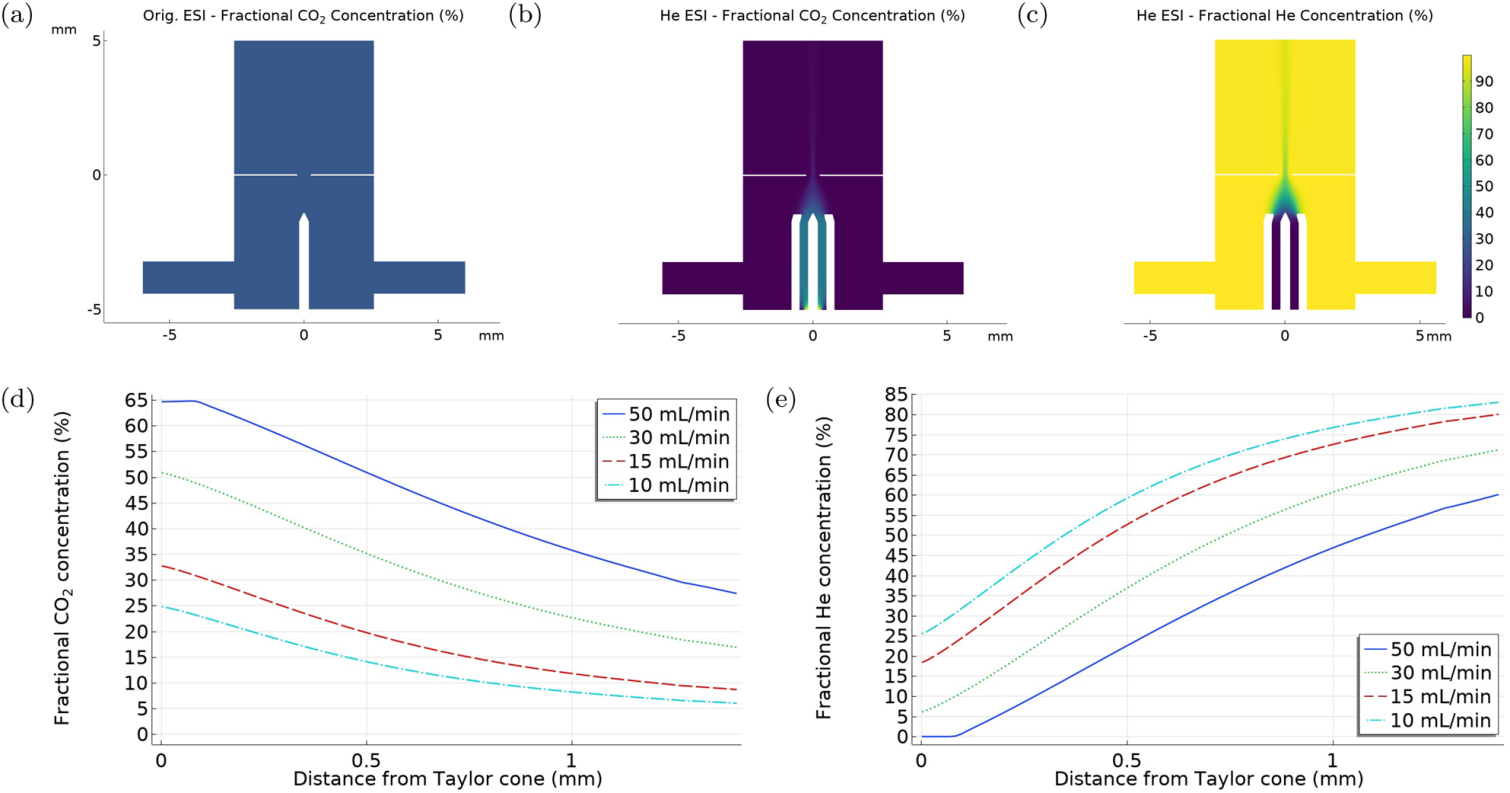
The fractional concentration of various gases in the vicinity of the Taylor cone, highlighting the effectiveness of gas shielding against corona discharge. (**a**) The fractional concentration of CO_2_ in the original ESI system. (**b,c**) The fractional concentration of CO_2_ and He respectively in the He-ESI system. (**d,e**) The fractional concentration of CO_2_ and He respectively in the He-ESI system under varying CO_2_ flow rates while maintaining a constant He flow rate of 1.2 L/min and N_2_ flow rate of 20 mL/min.

We compared the gas distribution between the original ESI and the He-ESI system, as displayed in Fig. 3a–c. In the original ESI system, a mixture of two gases was used. N_2_ was utilized as the carrier gas at a flow rate of 1 L/min, and CO_2_ was employed as a protective gas, to shield the Taylor cone from corona discharge, with a flow rate of 150 mL/min. The He-ESI system instead uses a mixture of three gases. He serves the role of the carrier gas with a flow rate of 1.2 L/min, while N_2_ and CO_2_, with flow rates of 20 mL/min and 15 mL/min respectively, functioned as protective gases. The simulation results illustrate how in the He-ESI system, the Taylor cone is effectively enveloped by CO_2_, preventing corona discharge.

To study the influence of various CO_2_ flow rates on the gas distribution around the Taylor cone in the He-ESI setup, we performed simulations at CO_2_ flow rates of 10, 15, 30, and 50 mL/min, as depicted in Fig. 3d, e . The He and N_2_ flow rates were kept constant at 1.2 L/min and 20 mL/min, respectively. These simulations help estimate the minimal fractional concentration of gases necessary to sustain a stable Taylor cone, thus minimizing the potential for corona discharge. This provides important insights into the interactions and flow dynamics of the gases around the Taylor cone, which can further help us optimize the design and operating conditions of the electrospray system.

### Operating conditions for the He-ESI

The He-ESI with the Uppsala nozzle is stable under the following conditions: the tip of the angled capillary (conically ground at an angle of 30^∘^) must be kept at the edge or slightly inside the nozzle, which is placed at a distance of 1.5–1.8 mm away from the grounded orifice of 0.5 mm diameter. The liquid sample flow rates must be 100–200 nL/min, the He flow rate in the ESI head should be 1.2–1.4 L/min, the N_2_ flow rate 0.03–0.035 L/min, the CO_2_ flow rate 0.015–0.02 L/min and the voltage between 2.2 and 2.6 kV. The stability of the He-ESI was monitored by measuring the current (typically around 200–300 nA) and visually with a camera pointing at the Taylor cone. Under these conditions, the Taylor cone ejects positively charged droplets that are electrostatically trapped on the grounded counter electrode^28^. For the sample particles to reach the interaction chamber it is thus essential to neutralize them. To achieve this, we passed them through a Po-210 neutralizer which functions by ionizing nearby gas molecules into positive and negative ions. The charged droplets are subsequently neutralized by capturing ions of the opposite charge, after which the solvent evaporates and leaves behind neutral sample particles. The particles are then transported through conductive tubing to the inlet of the experimental setup.

### Injector setup: operation using He-ESI

The He-ESI is coupled to the injector setup^17^. An extra helium inlet was added to the injector setup before the first skimmer stage to avoid the suction of the gas in the aerosolization and neutralization chamber of the ES due to the pumping in the skimmer stages and to protect the Taylor cone. Typically, 2.5–3 L/min He is added at the aerosol inlet. In total, 4.2 L/min He is required in the setup. The excess gas is skimmed away using scroll pumps at the two nozzle-skimmer stages. The particles enter the aerodynamic lens with a pressure of 1–1.2 mbar and exit the lens through a 1.5 mm aperture into the interaction region in the experimental chamber, which is kept at 10^-5^ mbar.

### Gas reduction in the interaction chamber

**Figure 4:**
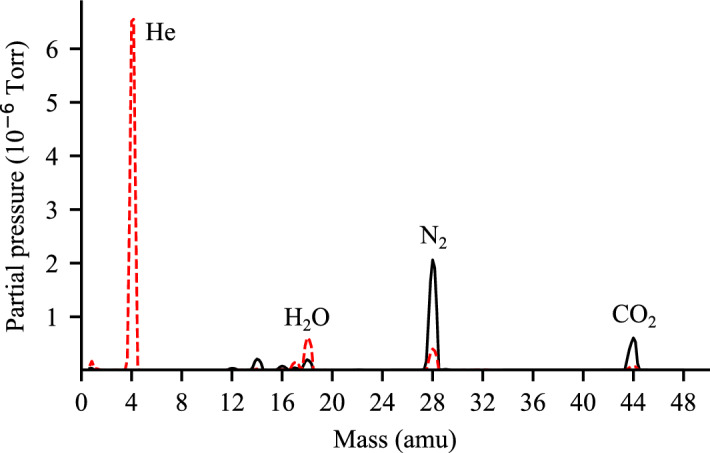
Residual gas analysis spectrum inside the interaction chamber. Measured for the He-ESI (dashed red) with flow rates of 4.2 L/min He, 0.03 L/min N_2_, 0.015 L/min CO_2_ and the original ESI (solid black) with flow rates of 1 L/min N_2_ and 0.2 L/min CO_2_.

We used an RGA, mounted 25 cm away from the interaction region, to determine the composition of the gas in the interaction chamber. RGA spectra while using both types of ESI are shown in Fig. 4. For the He-ESI (dashed red line), the largest contribution is He at 4 atomic mass units (amu) with a partial pressure, measured from the peak area, of $1.9\times 10^{-5}$ Torr, while N_2_ and CO_2_, shown in the spectrum at 28 and 44 amu, have partial pressures of $1.6\times 10^{-6}$ Torr and $3.1\times 10^{-7}$ Torr respectively. There’s a further peak at 18 amu due to water contamination.

The relative composition of the input gases to the He-ESI is 1.22% N_2_ and 0.97% CO_2_, compared to 8% N_2_ and 1.5% CO_2_ measured in the interaction chamber. This discrepancy may be explained by the different pumping efficiency for He, N_2_ and CO_2_ based on Graham’s law, which states that the rate of diffusion or effusion of gas is inversely proportional to its molecular weight. This implies, that N_2_ and CO_2_ diffuse much slower than He when passing through the nozzle in the two skimmer stages leading to He being skimmed away first and more efficiently.

The RGA spectrum of the original ESI, shown in black, shows much larger N_2_ and CO_2_ peaks, with partial pressures of $8.7\times 10^{-6}$ and $2.4\times 10^{-6}$ Torr respectively.

While the gases in the interaction chamber will scatter both elastically and inelastically, the inelastically scattered photons can be filtered due to their different energy. But the elastically scattered ones are indistinguishable from those scattered by the sample and are the main contributors to background noise in SPI^19^. For the resolutions relevant to SPI, each gas molecule is well approximated as a point scatterer and the total scattering is then proportional to the square of the number of electrons. We can then estimate the elastic scattering from the gas as the weighted sum of the contributions of the different gas species, and with it calculate the expected elastic scattering by the gas when using the He-ESI relative to the original ESI ($I_{rel}$),

$$I_{rel} = \frac{ p^{\text {new}}_{N_2} Z_{N_2}^2 + p^{\text {new}}_{CO_2} Z_{CO_2}^2 + p^{\text {new}}_{He} Z_{He}^2}{p^{\text {old}}_{N_2} Z_{N_2}^2 + p^{\text {old}}_{CO_2} Z_{CO_2}^2},$$

where $p^\text {new}$ are the partial pressures of the He-ESI setup, $p^\text {old}$ of the original ESI and *Z* is the total number of electrons of each gas molecule. Using this equation with the partial pressures measured above we obtain an $I_{rel}$ of 0.188 or an expected reduction of scattering intensity by $\approx 81$ %.

### Sample delivery performance with the He-ESI

To show that the He-ESI is working stable and generates aerosolized particles suitable for SPI experiments, we coupled the He-ESI to the “Uppsala”-injector. To detect the flow of particles into the interaction region we used a Rayleigh-scattering microscopy setup^24^ and recorded particle intensities and beam evolution curves for 20–80 nm polystyrene spheres (PS) in a 20 mM ammonium acetate (AmAc) buffer solution. The beam-evolution curve is shown in the supplementary information Fig. S2. At a given injector pressure, the particle-beam focus position moves away from the injector exit with increasing particle size. A similar behaviour has been observed in a previous study using the same injector and GDVN aerosolization, i.e. focusing with He, for PS with diameters larger than 40 nm^4^. For larger particles, the gas density in the focus is lower. In addition, the opening angle of the particle beam decreases with increasing particle size. The particle-beam parameters are summarized in Table S1. The data collection and analysis are discussed in^24^. Next, we characterized the particle-beam density at the particle beam focus for different sizes of PS and Bacteriophage MS2 (MS2) and compared it to particle-beam density measurements using the original ESI for aerosolization, i.e. using N_2_ as the main carrier gas. As a proxy for the particle-beam number density, we show the number of particles collected in 1000 frames. Each frame contains one laser pulse for particle detection. Table 1 shows the measured mean number of particles detected per 1000 frames in the particle-beam focus. For all particle sizes, the measured number of particles is higher using the He-ESI compared to the original ESI. While the improvement of the measured particle numbers is different for the used sizes, the highest improvement in particle throughput, by a factor of $\approx$ 11, is observed in the bioparticle MS2.

**Table 1:** Comparison between He-ESI and original ESI of the mean number of particles per 1000 frames as a function of the sample diameter.

Sample/DMA size (nm)	Particles per 1000 frames	
	He-ESI	Original-ESI
Bacteriophage MS2/ 25.9	460	42
20 nm PS/ 18.9	1010	271
30 nm PS/ 28.9	2546	517
40 nm PS/ 42.9	2264	874
50 nm PS/ 59.4	1553	939
70 nm PS/ 76.4	1118	527
80 nm PS/ 88.2	1150	300

#### Exploration of Various Ionization Techniques in He-ESI

A comparative study was conducted to analyze the transmission efficiency between two different neutralization techniques used in He-ESI: a Polonium (Po-210) source, a radioactive alpha particle emitter, and a vacuum ultraviolet (VUV) ionizer, which is essentially a VUV deuterium lamp. Both neutralizers function by ionizing nearby gas molecules into positive and negative ions, enabling ES-generated charged particles to be neutralized by capturing ions of the opposite charge. The key difference lies in their ionization methods: the Po-210 source ionizes gas molecules through collisions with alpha particles, whereas the VUV ionizer employs photoionization to achieve the same effect. The target sample utilized for this experiment was cube-shaped silver nanoparticles, with a side length of 75 nm, suspended in ethanol. In both techniques, the gas flow rates were maintained at 1 L/min for He and 30 mL/min for CO_2_. Particle detection was carried out in the interaction chamber using Rayleigh scattering^24^. The results from the number of particles detected showed that the Polonium source delivered approximately 5% more particles than the VUV ionizer to the interaction chamber.

Nonetheless, given that VUV light is more efficient at ionizing N_2_ gas^16^, we extended our experiment by adding 30 mL/min of N_2_ through the He inlet. This introduction of N_2_ enhanced the transmission efficiency of the VUV ionizer setup and outperformed the Polonium source setup by delivering approximately 30% more particles. This enhancement can be attributed to the improved neutralization of the particles, facilitated by the VUV ionizer’s more effective in ionizing N_2_. These findings suggest that the inclusion of N_2_ gas in the VUV ionizer setup could be a potential strategy to enhance transmission efficiency in He-ESI. It is important to highlight, though, that the advantages gained from incorporating N_2_ need to be balanced against its potential contribution to background noise.

## Discussion and outlook

Within this paper, we presented improvements in the sample aerosolization process by developing a He-ESI to reduce the background scattering due to gases in SPI experiments. We used 3D-printed nozzles to reduce the amount of N_2_ and CO_2_ and kept modifications of the previously used ESI setup to a minimum. With the He-ESI, the main particle transport gas into the interaction chamber is He. In the interaction chamber and based on RGA measurements using the He-ESI with the Uppsala-nozzle, the amount of N_2_ was reduced by 82 % and for CO_2_ by 87.7 %. While the large reduction of the heavy gasses in the initial gas mixture could not be observed to the same extent in the interaction region, presumably due to different pumping efficiencies, an optimization of the skimmer assembly may improve the ratio in the interaction region further. Nonetheless, assuming the ratio of the gasses measured in the RGA translates into the ratio of contribution to background scattering, we reduced the gas background scattering off the gas by 81 %.

Additionally, through simulations conducted using COMSOL Multiphysics, our study has deepened the understanding of gas flow dynamics around the Taylor cone in a He-ESI system. This allowed us to model the behaviour of different gas mixtures, examining their respective impacts on protecting the Taylor cone from corona discharge. Given our optimal operational conditions with a water-based buffer our simulations suggest that to maintain a stable Taylor cone, the He percentage should not exceed 20% at the cone’s tip. Further computational analysis of the gas distribution and breakdown voltage can aid in determining the minimum fractional concentration necessary to maintain a stable cone before corona discharge occurs.

Our modification of the ESI not only demonstrates a decreased use of heavy gasses for sample injection but also an increased throughput of particles into the interaction region. The highest increase in transmission of particles was observed while injecting small bioparticles: approximately by a factor of 11 for MS2 particles. Whereas, while delivering PS into the interaction region, we measure an increase in the transmission of particles by a factor of 2 to 5 depending on the particle size.

To further enhance particle transmission, we conducted a comparative analysis of the transmission efficiency between Po-210 sources and VUV ionizer techniques within He-ESI systems. Our results demonstrated that by adding 30 mL/min of N_2_ gas along with He at the He inlet, the VUV ionizer’s performance was enhanced, surpassing the Po-210 source by approximately 30%. For a more comprehensive understanding of their impact on transmission efficiency, future studies could investigate the simultaneous utilization of both the Po-210 source and the VUV ionizer in He-ESI systems.

Although this is not the first adaptation of ES injection for X-ray diffractive imaging, the presented modification is a much-required leap towards single protein imaging by aiming at lower background scattering from the injection gases, allowing us to recognize lower scattering signals from the sample in the diffraction data. We expect the He-ESI to improve the quality of collected data and provide better experimental conditions for X-ray imaging of small nanoparticles not only due to the lowered background but also because of a higher particle transmission through the injector. Together, higher quality and quantity of diffraction patterns can be collected in the future using a He-ESI for sample aerosolization.

### Supplementary Information

Supplementary Information.

## Data Availability

The data presented in this study are available upon request from the corresponding author.

## References

[1] Neutze R, Wouts R, Van der Spoel D, Weckert E, Hajdu J (2000). Potential for biomolecular imaging with femtosecond X-ray pulses. Nature.

[2] Miao, J., Ishikawa, T., Robinson, I. K. & Murnane, M. M. Beyond crystallography: Diffractive imaging using coherent X-ray light sources. *Science* **348**, 530–535 (2015). https://www.science.org/doi/abs/10.1126/science.aaa1394. 10.1126/science.aaa1394 25931551

[3] Aquila, A. *et al.* The linac coherent light source single particle imaging road map. *Struct. Dyn.* **2** (2015). 10.1063/1.4918726 PMC4711616 26798801

[4] Hantke MF (2018). Rayleigh-scattering microscopy for tracking and sizing nanoparticles in focused aerosol beams. IUCrJ.

[5] Seibert MM (2011). Single mimivirus particles intercepted and imaged with an X-ray laser. Nature.

[6] Ekeberg T (2015). Three-dimensional reconstruction of the giant mimivirus particle with an X-ray free-electron laser. Phys. Rev. Lett..

[7] Munke A (2016). Coherent diffraction of single rice dwarf virus particles using hard X-rays at the linac coherent light source. Sci. Data.

[8] Reddy HK (2017). Coherent soft X-ray diffraction imaging of coliphage pr772 at the linac coherent light source. Sci. Data.

[9] Lundholm IV (2018). Considerations for three-dimensional image reconstruction from experimental data in coherent diffractive imaging. IUCrJ.

[10] Rose M (2018). Single-particle imaging without symmetry constraints at an X-ray free-electron laser. IUCrJ.

[11] Hantke MF (2014). High-throughput imaging of heterogeneous cell organelles with an X-ray laser. Nat. Photon..

[12] Van Der Schot G (2015). Imaging single cells in a beam of live cyanobacteria with an X-ray laser. Nat. Commun..

[13] Ayyer, K. *et al.* 3d diffractive imaging of nanoparticle ensembles using an X-ray laser. *Optica* **8**, 15–23 (2021). https://opg.optica.org/optica/abstract.cfm?URI=optica-8-1-15.

[14] Bogan MJ (2008). Single particle X-ray diffractive imaging. Nano Lett..

[15] Worbs L (2021). Optimizing the geometry of aerodynamic lens injectors for single-particle coherent diffractive imaging of gold nanoparticles. J. Appl. Crystallogr..

[16] Rafie-Zinedine S (2024). Enhancing electrospray ionization efficiency for particle transmission through an aerodynamic lens stack. J. Synchrotron Rad..

[17] Bielecki, J. *et al.* Electrospray sample injection for single-particle imaging with X-ray lasers. *Sci. Adv.* (2019). 10.1126/sciadv.aav8801 PMC6499549 31058226

[18] Bellisario A, Maia FR, Ekeberg T (2022). Noise reduction and mask removal neural network for X-ray single-particle imaging. J. Appl. Crystallogr..

[19] Ekeberg, T. *et al.* Observation of a single protein by ultrafast X-ray diffraction. *bioRxiv* 2022-03 (2022). 10.1038/s41377-023-01352-7 PMC10786860 38216563

[20] Pandey S (2020). Time-resolved serial femtosecond crystallography at the European xfel. Nat. Methods.

[21] Poudyal I, Schmidt M, Schwander P (2020). Single-particle imaging by X-ray free-electron lasers—How many snapshots are needed?. Struct. Dyn..

[22] Bielecki J, Maia FRNC, Mancuso AP (2020). Perspectives on single particle imaging with X rays at the advent of high repetition rate X-ray free electron laser sources. Struct. Dyn..

[23] Chen, D.-R., Pui, D. Y. & Kaufman, S. L. Electrospraying of conducting liquids for monodisperse aerosol generation in the 4 nm to 1.8 Ã‚Âµm diameter range. *J. Aerosol Sci.* (1995).

[24] Yenupuri, T. V., Worbs, L., You, T. & Maia, F. R. N. C. Optical detection and sizing of single sub-20 nm bioparticles in a focused aerosol particle beam (2024) (manuscript in preparation).

[25] Zeleny, J. The electrical discharge from liquid points, and a hydrostatic method of measuring the electric intensity at their surfaces. *Phys. Rev.* **3**, 69–91 (1914). 10.1103/PhysRev.3.69.

[26] Taylor, G. I. S. Disintegration of water drops in an electric field. *Proc. R. Soc. Lond. Ser. A Math. Phys. Sci.* **280**, 383–397 (1964).

[27] COMSOL. Comsol Multiphysics®. http://www.comsol.com (2022).

[28] Huijing Fu MJH, Patel AC, Chen D-R (2011). A new electrospray aerosol generator with high particle transmission efficiency. Aerosol Sci. Technol..

