# Supplementary material for: Helium-electrospray improves sample delivery in X-ray single-particle imaging experiments
Source: Sci Rep. 2024 Feb 22;14:4401. doi: 10.1038/s41598-024-54605-9 (PMC10883998; doi:10.1038/s41598-024-54605-9)
Supplement: Supplementary file 1 — Supplementary Information. [file 41598_2024_54605_MOESM1_ESM.pdf]

Supplementary Materials for: Helium-electrospray improves sample delivery in X-ray single-particle imaging experiments

Tej Varma Yenupuri[‡1], Safi Rafie-Zinedine[‡2,3], Lena Worbs[1], Michael Heymann[3], Joachim Schulz[2], Johan Bielecki[*2], and Filipe R. N. C. Maia[*1,4]

[1]*Laboratory of Molecular Biophysics, Department of Cell and Molecular Biology, Uppsala University, Husargatan 3 (Box 596), Uppsala, 75124, Sweden*

[2]*European XFEL, Holzkoppel 4, 22869 Schenefeld, Germany*

[3]*Institute of Biomaterials and Biomolecular Systems, University of Stuttgart, Pfaffenwaldring 57, Stuttgart, 70569, Germany*

[4]*Lawrence Berkeley National Laboratory, Berkeley, CA, 94720, USA*

[‡]These authors contributed equally to this work.

[*] Correspondence e-mail: johan.bielecki@xfel.eu, filipe.maia@icm.uu.se

1 METHODS AND RESULTS

1.1 He-ESI Design: the EuXFEL Nozzle

The EuXFEL nozzle was engineered using Siemens' NX software and was designed with three capillary inlets suitable for 360 µm outer diameter (OD) fused silica capillaries for fluid feed, along with two outlets as shown in Figure 1. The inlet ports comprised one for a sample with an inner diameter (ID) of 40 µm, another for gas with an ID of 180 µm, and a dummy one which aids in centering the sample capillary. The outlet ports included one designated for the sample, with an ID of 40 µm and an angle of approximately 10°, and another, concentric with the first, designated for gas, with an ID of 410 µm and an angle of approximately 7°.

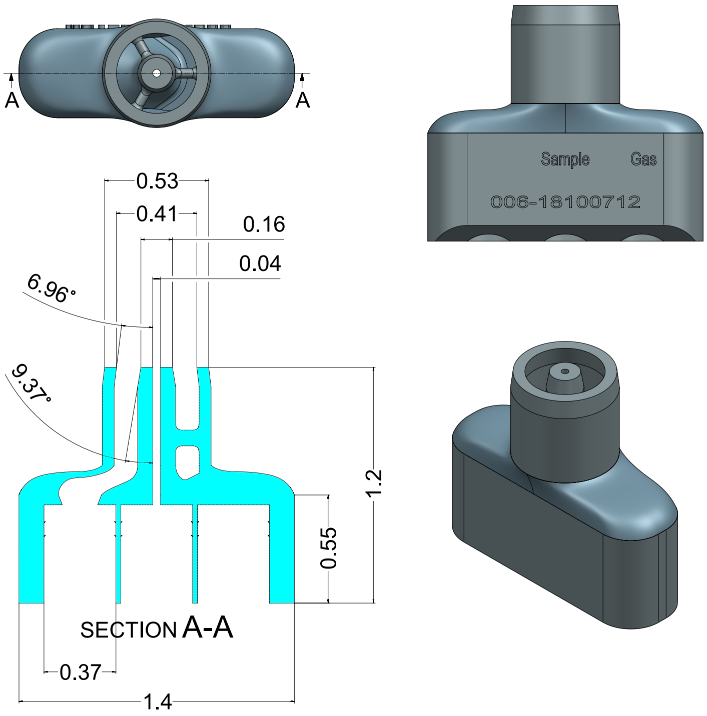

Figure 1: Schematic drawings of the EuXFEL Nozzle illustrate the dimensions and depict the inlets and outlets of the nozzle.

The nozzle design was outputted in STL formats. The conversion of these STL-based 3D designs into print-job instructions, or GWL, was executed using Nanoscribe's DeScribe software. For better structural stability of the fabricated devices, we adopted a solid volume printing strategy with slicing of 1 µm and hatching of 0.5 µm. The devices were then printed using the Nanoscribe Photonic Professional GT with IP-S photoresist as the printing material. The process utilized a 25x objective lens from Zeiss, full laser power, and a printing velocity of 100.000 $\mu m\, s^{-1}$. Under these conditions, the printing duration for a single device was approximately one hour.

Following the printing process, the glass slide with the cured photoresist was submerged in a beaker of propylene glycol methyl ether acetate (PGMEA) for one or two days to dissolve any remaining uncured parts, a process known as development. Post-development, the devices were transferred to a beaker of isopropanol for about 30 min, then relocated to another beaker filled with fresh isopropanol. Finally, the devices were left on a cleanroom cloth to dry under ambient conditions.

The nozzles were assembled on clean polydimethylsiloxane (PDMS) sheet, with the process monitored under an optical microscope. To secure the devices, an additional piece of PDMS was applied over them. Following this, three fused silica capillaries, each with an OD of 360 µm, were inserted into their designated fluid inlets on the nozzle and secured with a 5-minute epoxy glue from Devcom. These capillaries were then guided through hollow stainless-steel tubing with an OD of 1/16 inch (IDEX U-145 with an ID of 0.046 inches) and glued between the nozzle material and steel.

1.2 Operating Conditions for the EuXFEL Nozzle

The operating stability of the He-ESI system is influenced by factors such as the buffer type, the buffer conductivity, and the geometry of the aerosolization chamber. To minimize the presence of heavier gases

and maintain a stable Taylor cone, the operating conditions were carefully optimized. Experiments were conducted using the EuXFEL nozzle with two different buffers: water (with conductivities ranging from 900 to 1600 μS/cm) and ethanol (with conductivities ranging from 800 to 1300 μS/cm). With the water buffer, we used a He flow rate of $1 - 1.5$ L/min, a N_2 flow rate of $20 - 30$ mL/min, and a CO_2 flow rate of $15 - 25$ mL/min. With the ethanol buffer, the He flow rate was adjusted to $1 - 1.6$ L/min, while the CO_2 flow rate was set at $10 - 20$ mL/min, without any N_2 flow. The nozzle was tested with two different ionizers: a Po-210 source and a UV ionizer, before transporting the particles to the Uppsala injector.

1.3 PS particle-beam parameters

The particle-beam width depending on the distance from the injector exit was measured for different sizes of PS. A Gaussian beam evolution fit was used to determine the focus width and the focus position. The particle-beam evolution curves are shown in Figure 2 and the focus values are summarized in Table 1. A clear shift of the particle-beam focus towards the injector exit with decreasing particle size is observed and the particle-beam focus width increases as the particle size decreases.

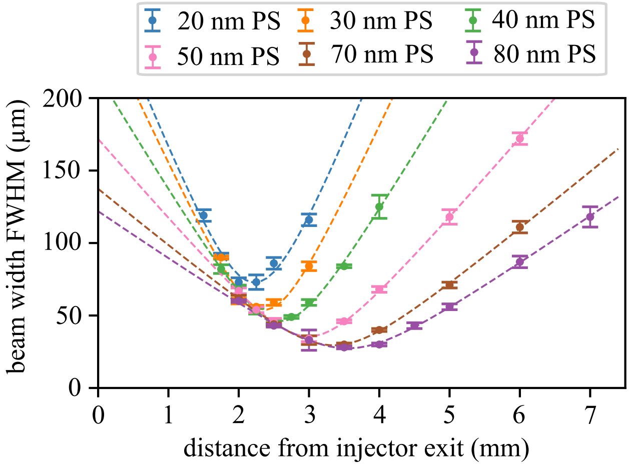

Figure 2: Particle-beam evolution curves for 20 - 80 nm PS at 1.0 mbar injector pressure using the He-ESI with Uppsala nozzle for aerosolization. The main focussing gas is He.

Sample/DMA size (nm)	focus position (mm)	focus width FWHM (μm)
20 nm PS/ 18.9	2.23	73
30 nm PS/ 28.9	2.37	54
40 nm PS/ 42.9	2.59	46
50 nm PS/ 59.4	2.99	36
70 nm PS/ 76.4	3.35	29
80 nm PS/ 88.2	3.55	27

Table 1: Experimental particle-beam parameters (focus position and width) for different sizes of PS. The particles were aerosolized using the He-ESI and the injector pressure was kept constant at 1.1 mbar.
